# Preferences for end-of-life care in geriatric trauma surgery patients with immobilising fractures – a prospective cohort study

**DOI:** 10.1186/s12877-026-07047-z

**Published:** 2026-01-29

**Authors:** Vanessa Ketter, Robin Janine Ahles, Nils Heuser, Julia Lenz, Anton Korschinsky, Steffen Ruchholtz, Christian Volberg

**Affiliations:** 1 Center for Orthopaedics and Trauma Surgery, Philipps University of Marburg, Marburg, Germany; 2 Helios Kliniken Kassel, Kassel, Germany; 3 Department of Anesthesiology and Intensive Care Medicine, Philipps University of Marburg, Baldingerstraße, Marburg, 35043 Germany

**Keywords:** End-of-life care, Trauma surgery, Geriatric medicine, Immobilisation, Advance directive, Last phase of life

## Abstract

**Background:**

The risk of immobilising fractures is increased in elderly individuals. This acute event, combined with subsequent bedriddenness, has been shown to increase mortality by approximately 24% within one year. Despite advanced age, conversations with relatives or treating physicians about the last phase of life are rarely held.

The objective of this study is to examine the extent to which elderly individuals have addressed their own end-of-life preferences and made suitable provisions.

**Methods:**

A prospective cohort study was conducted in a supraregional trauma centre. Recruitment occurred from May 2023 to May 2024. A descriptive data analysis was performed using a specially designed questionnaire with 25 questions. In addition to demographic parameters, the questionnaire included parameters such as the existence of an advance directive, health care discussions held in the clinical setting, and the preferred place of death.

**Results:**

A total of 138 patients were screened, of whom 51 could be included in the study. The study revealed that a mere 8% (n=4) of respondents had engaged in a discussion regarding preventive care during their initial admission to the clinic. However, it was observed that 51% (n=26) of patients expressed a willingness to participate in such a discussion and that their recent fracture had a direct impact on their inclination to seek preventive consultation.

Furthermore, 65% (n=33) of those surveyed expressed a desire to die at home, although 67% (n=22) of them had not disclosed this wish to anyone.

The data indicates that 59% (n=30) of individuals have already designated an advance directive and health care proxy, though these documents frequently entail only general wording and have not been thoroughly discussed.

**Conclusion:**

A significant proportion of patients have already given thought to the place they would prefer to die but without engaging in dialogue with others about this wish. Considering these findings, it is incumbent upon the treating physicians and nursing staff to initiate discussions concerning early care planning, thereby facilitating the articulation of patients' wishes in acute palliative scenarios. This approach ensures that these wishes are duly considered and acted upon.

**Trial registration:**

German Clinical Trials Register (DRKS00031589), Registration date: 03 April 2023.

## Introduction

The current average life expectancy in Germany is 78.2 years for men and 82.9 years for women [1]. In addition to internal diseases, the risk of accidents, such as falls, increases with age. Proximal femur fractures (e.g. femoral neck fractures or pertrochanteric fractures) are a typical fracture of the elderly, the incidence of which increases sharply with age (2550/100,000 for pertrochanteric fractures in women > 90 years) [2]. The in-hospital mortality rate for immobilising fractures in geriatric patients ranges from 5.5 to 8.3%, rising to 23.9% in the year following the accident [3]. The prognosis is particularly poor in patients with pre-existing conditions or perioperative complications (e.g. pneumonia, renal failure) [4]. Several studies have already indicated an impending increase in such fractures, with a predicted escalation in the coming years [5]. This underlines the need to consider patient preferences.

Surveys have revealed a societal reluctance to speak about death and dying, compounded by a persistent lack of advance directives. It is clear that both patients and physicians initiate these discussions far too infrequently [6].

Representative population surveys show that the majority of Germans would like to die at home (59–66%) [7]. However, the reality is that only 21% of the population die at home, with the majority (over 50%) dying in hospital or in a nursing home (around 20%) [8]. Despite this knowledge, end-of-life discussions often do not take place and patients are often “over-treated” at the end of life [9]. This means that dying cannot take place according to the patient’s wishes.

The concept of advance care planning is gaining prominence, not only in response to legislative developments, such as the German Hospice and Palliative Care Act, but also as a result of wider public discourse [10].

There is a paucity of research on patients’ wishes at the end of life, and the prevalence of precautionary documents remains inadequate. The present study investigates the wishes of patients with immobilising fractures regarding end-of-life care and their preferred place of death and the influence of the acute event on these wishes. The study had two objectives: strengthening patient autonomy and highlighting overtreatment at the end of life.

## Materials and methods

The present study was submitted to the institutional review board of the Department of Human Medicine at the Philipps University of Marburg for review (file number: 23/62 BO) and subsequently registered in the German Clinical Trials Register (DRKS00031589).

### Data collection

Following the positive vote, this prospective cohort study was conducted at the supra-regional trauma and geriatric trauma centre of the University Hospital of Marburg, Germany, from 02.05.2023–30.04.2024.

Patients were included in the study if they met the following criteria:

- an acute (temporary) immobilising fracture, such as a proximal femur fracture, a pelvic ring fracture, a periprosthetic knee or hip fracture, or a vertebral body fracture.
- age ≥ 80 years.
- sufficient knowledge of German and consent to participate in the study.

Patients were included in the study either postoperatively or at any point during their inpatient treatment if no surgical treatment had taken place. After providing information and consent, the study questionnaire was administered and completed.

The questionnaire contained a total of 25 questions, asking about demographic and disease-related data, as well as about the respondent’s involvement in care planning for the end of life and communication with relatives and treating physicians about it.

The individual sections covered the following dimensions:

- the extent of the subject’s knowledge about their own illness
- the subject’s preferences regarding the preferred place of death
- the preparation of documents relating to advance medical directives
- the way in which the patient has communicated their wishes for the last phase of life, and how these wishes have been handled.

The questionnaire for this study was adapted from questionnaires used in three previous studies that investigated the same topic in dermato-oncology, uro-oncology and dialysis patients [11–13]. The study team adapted the questionnaire for this study to the field of traumatology. The order of the questions was carefully designed to ensure that respondents found it easy to answer. Questions about personal history and current circumstances were asked first, followed by questions about advance directives, health care proxies, and thoughts about end-of-life wishes and the preferred place of death.

### Statistical analysis

Microsoft^®^ Excel version 16.68 was used for data analysis and management. For descriptive analyses, categorical data are presented as counts and percentages, while continuous variables are presented as mean with standard deviation (SD) or median with interquartile range (IQR) in the case of a skewed distribution.

The STROBE (Strengthening the Reporting of Observational Studies in Epidemiology) guidelines for cohort studies were used as a reporting checklist when creating the manuscript.

## Results

A total of 138 patients with a minimum age of over 80 years were screened, of whom 37% (*n* = 51) could be included in the study. The mean age of the sample was 86 ± 4.45 years, and 25.5% (*n* = 13) of the patients were female. In addition, 88.2% (*n* = 45) had an ASA score of 3 or 4. Nearly half of the patients (47,1%, *n* = 24) had a level of care of 3 or higher and were dependent on assistance.

Other demographic data and the type of immobilising fracture are shown in Table 1.

**Table 1:** Demographic data (ASA classification = American society of anesthesiologists physical status classification; level of care = A care level is a German classification that assesses the degree to which a person’s independence is impaired in six areas of life, to determine their entitlement to benefits from German care insurance)

Total [*n*]	51
Age [y]	86 ± 4.45
Gender [%]	
m	25.5 (*n* = 13)
f	74.5 (*n* = 38)
ASA [%]	
II	11.8 (*n* = 6)
III	84.3 (*n* = 43)
IV	3.9 (*n* = 2)
Marital Status [%]	
Married	62.7 (*n* = 32)
Widowed	31.4 (*n* = 16)
Divorced, Single	3.9 (*n* = 2)
Divorced, Permanent Relationship	2 (*n* = 1)
Living Situation [%]	
Own Home	84.3 (*n* = 43)
Nursing Home	3.9 (*n* = 2)
Assisted Living	5.9 (*n* = 3)
Childrens‘ Home	5.9% (*n* = 3)
Level of care [%]	
1	2 (*n* = 1)
2	27.5 (*n* = 14)
3	37.2 (*n* = 19)
4	7.8 (*n* = 4)
5	2 (*n* = 1)
None	23.5 (*n* = 12)
Assistance [%]	
Relatives	27.5 (*n* = 14)
Outpatient Nursing Service	35.3 (*n* = 18)
Nursing Home	3.9 (*n* = 2)
Personal Caregiver	2 (*n* = 1)
None	31.4 (*n* = 16)
Type of Fracture [%]	
Femoral Neck	23.5 (*n* = 12)
Pertrochanteric	15.7 (*n* = 8)
Vertebral Body	15.7 (*n* = 8)
Pubic Bone	7.8 (*n* = 4)
Pelvic Ring	7.8 (*n* = 4)
Subtrochanteric	5.9 (*n* = 3)
Acetabulum	5.9 (*n* = 3)
Total Hip Arthroplasty	3.9 (*n* = 2)
Greater Trochanter	2 (*n* = 1)
Periprosthetic:	
* Knee*	2 (*n* = 1)
* Hip*	2 (*n* = 1)
* Femur*	11.8 (*n* = 6)

Of all patients screened, 63% (n = 87) patients were deemed ineligible for inclusion in the study for reasons including the presence of dementia, communication barriers, unwillingness to participate, language discrepancies or postoperative delirium (see Table 2).

**Table 2:** Patients excluded from analysis

	*n*
Total	87
Age [y]	87.5 ± 4.9
Reasons for exclusion [%]	
Dementia	41.4 (*n* = 36)
No communication possible (e.g. hearing loss)	34.5 (*n* = 30)
No consent	14.9 (*n* = 13)
Post-operative delirium	4.6 (*n* = 4)
Language barrier	3.4 (*n* = 3)
Incapable of giving consent	1.1 (*n* = 1)

### Advance care documents

**Fig. 1:**
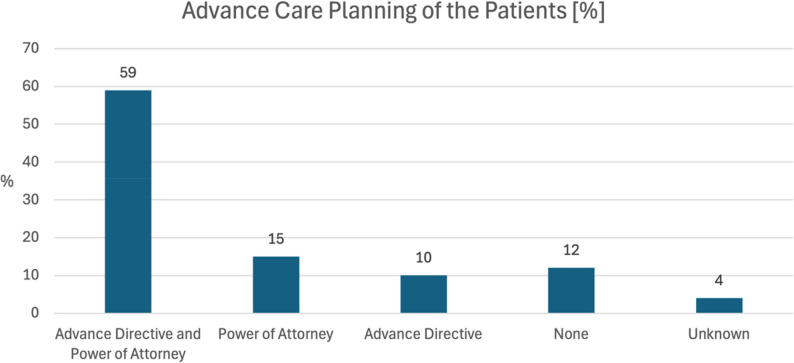
Advance care planning of the patients

Most patients included in the study (84.3%) had already completed some form of advance care planning, most commonly an advance directive and/or a power of attorney. Of these, 59% (*n* = 30) patients had both an advanced directive and a power of attorney, 10% (*n* = 5) patients had only an advance directive, and 15% (*n* = 8) patients had a power of attorney. 12% (*n* = 6) patients had not yet drawn up an advance care plan, and 4% (*n* = 2) patients were unaware of it, as shown in Fig. 1.

### Thoughts and wishes for the end of life

**Fig. 2:**
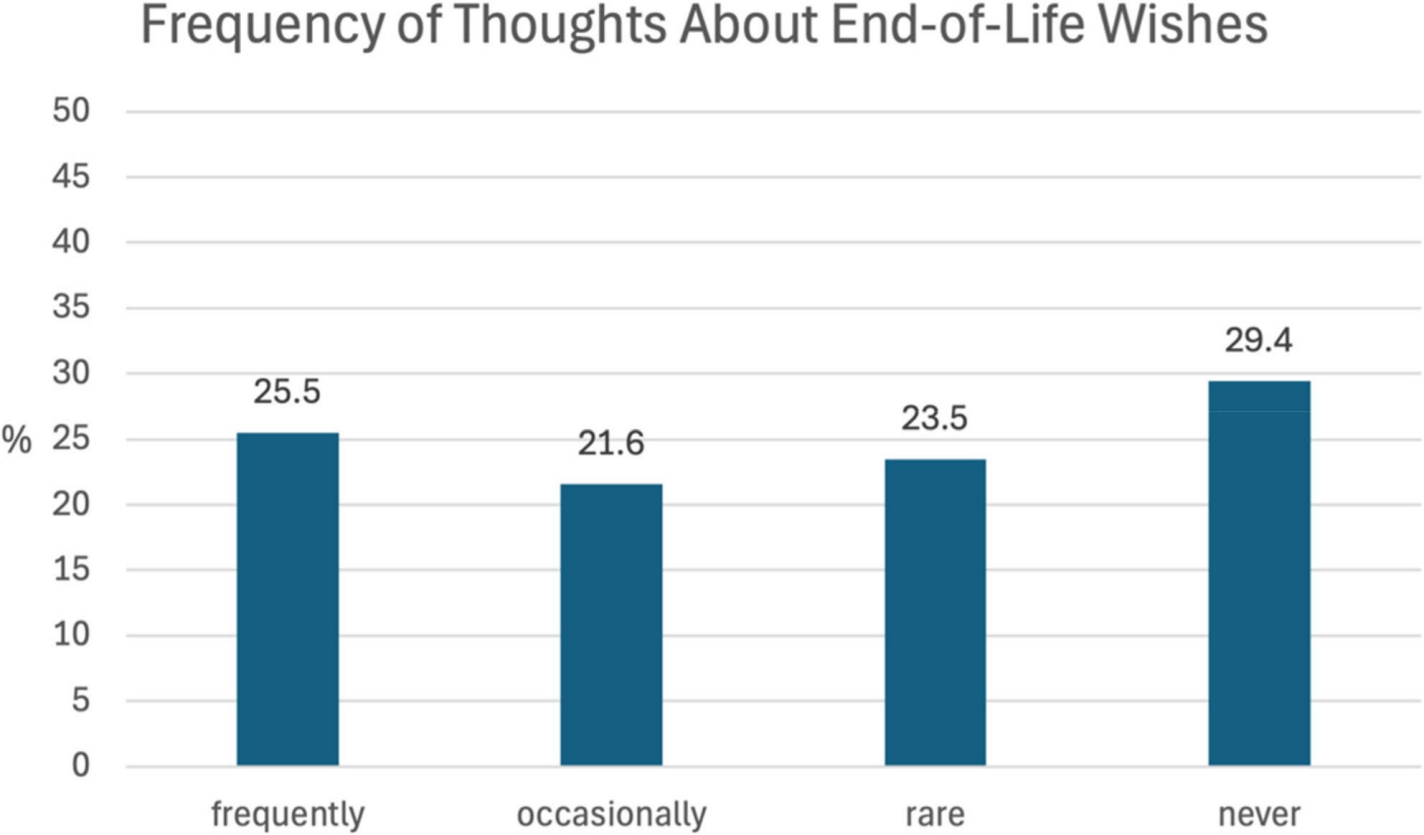
Frequency of thoughts about wishes for the end of life

Patients were asked how often they think about their preferences for end-of-life care. The results show that 47% (*n* = 24) patients had frequently or occasionally thought about this, while 53% (*n* = 27) patients had rarely or never done so, as shown in Fig. 2.

**Fig. 3:**
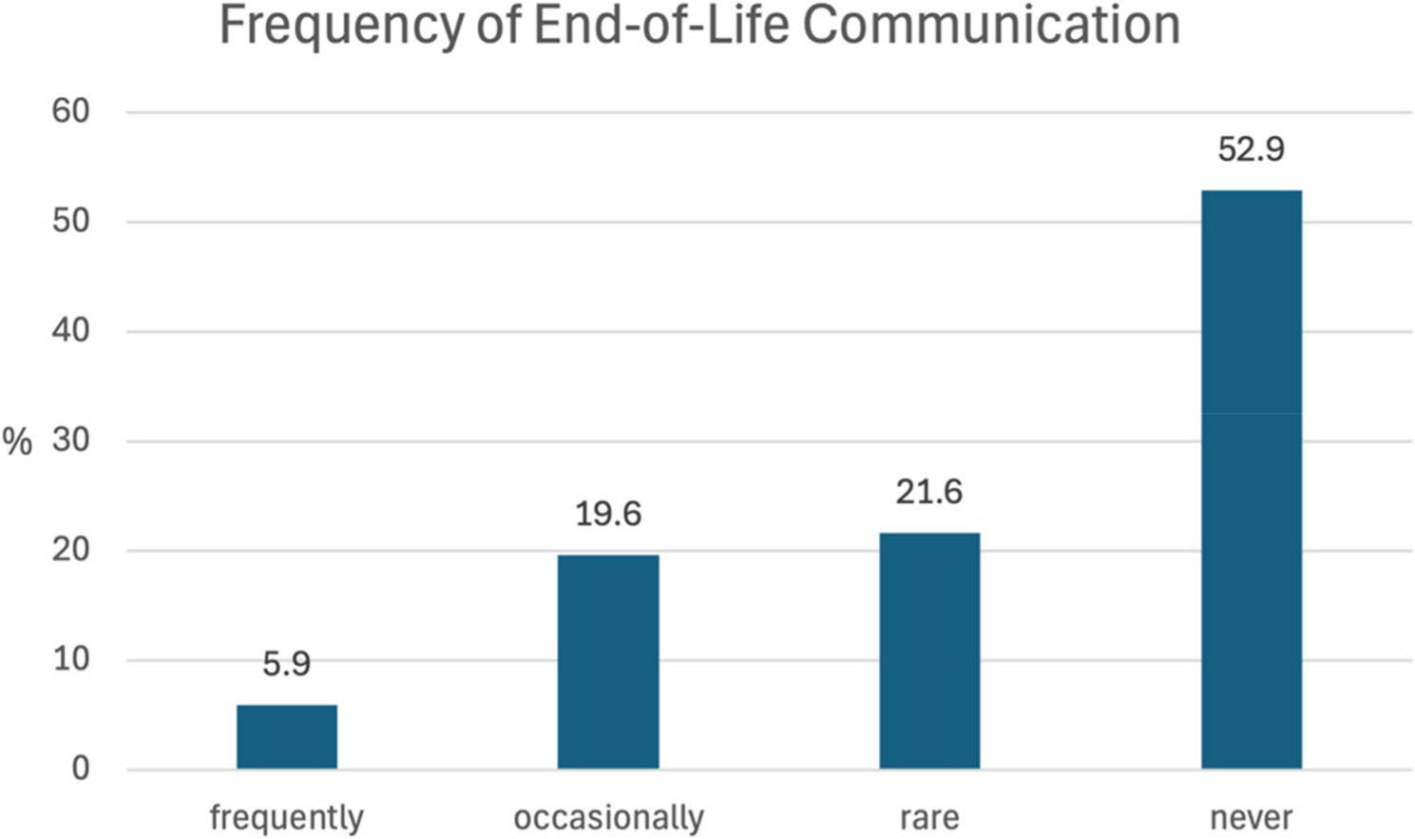
Frequency of communication about end-of-Life wishes

**Fig. 4:**
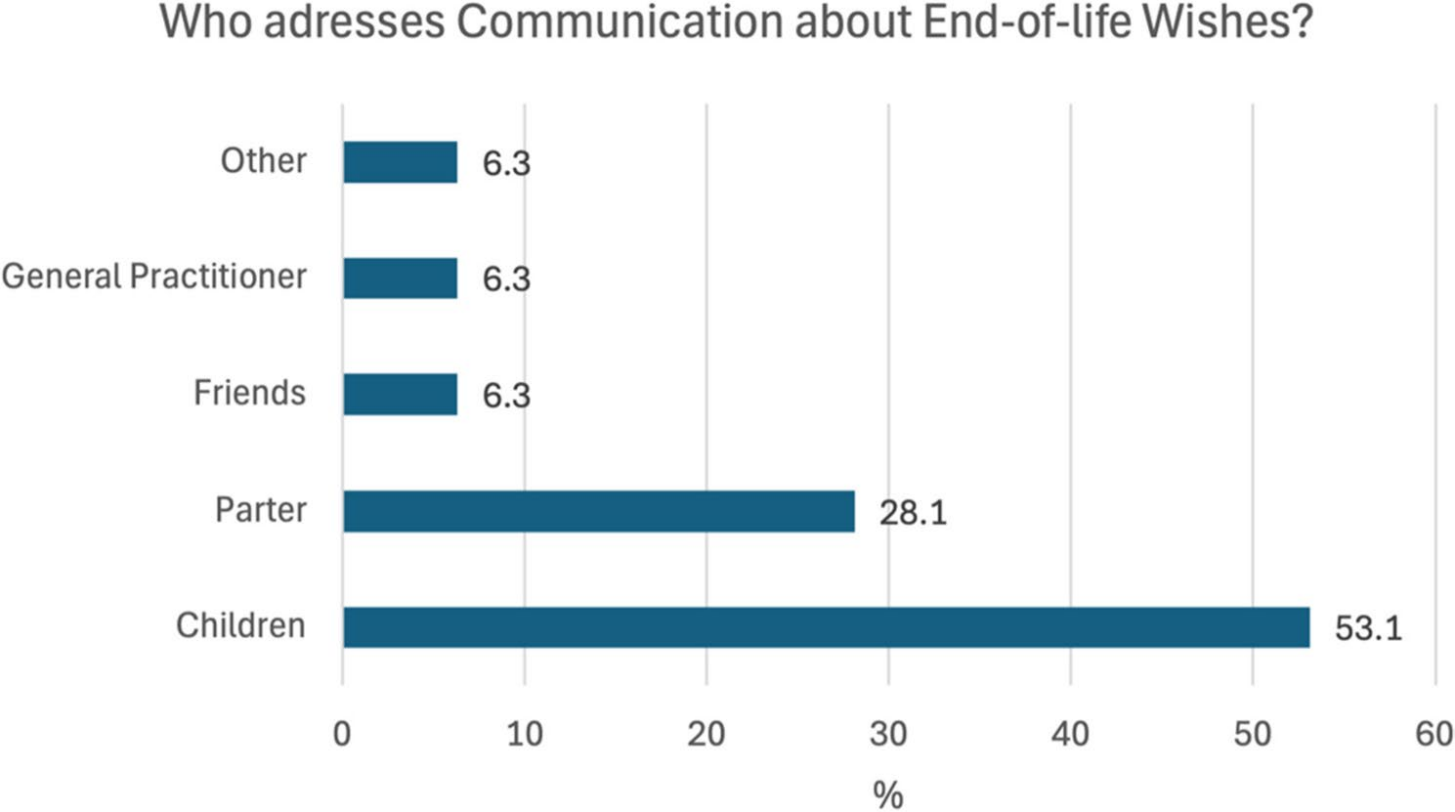
Who addresses communication about wishes for the end of life?

In addition, 25.5% (*n* = 13) patients reported that they were frequently or occasionally asked about their wishes for the end of life, with the majority of these cases being prompted by their children. 74.5% (*n* = 38) patients were rarely or never asked about their wishes (see Figs. 3 and 4).

### Preferred place of death

**Fig. 5:**
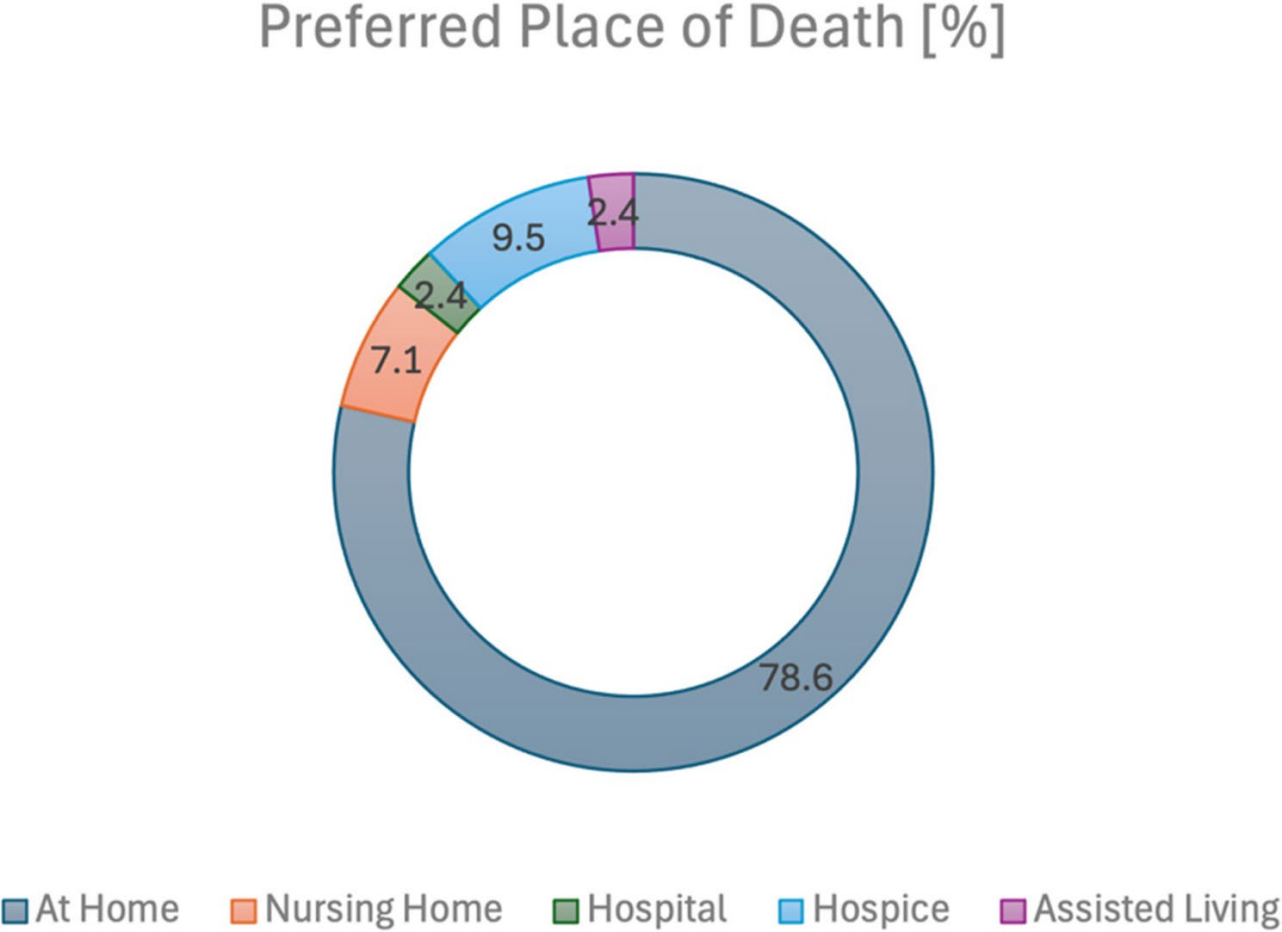
Preferred place of death

All patients were asked if they have a preferred place of death. 17.7% (*n* = 9) said that this was not important to them. The remaining 82.4% (*n* = 42) expressed a specific preference. The majority of these patients wished to die at home (78.6%; *n* = 33). The next most popular choice was a hospice (9.5%; *n* = 4), followed by a nursing home (7.1%; *n* = 3) (see Fig. 5).

Of the participating patients, 57% (*n* = 29) told their relatives about their wishes regarding their preferred place of death, while 43% (*n* = 22) did not discuss their preferences with anyone. Only two patients (4%) documented their preferred place of death in precautionary documents.

### Conversations regarding end-of-life care in trauma surgery

**Fig. 6:**
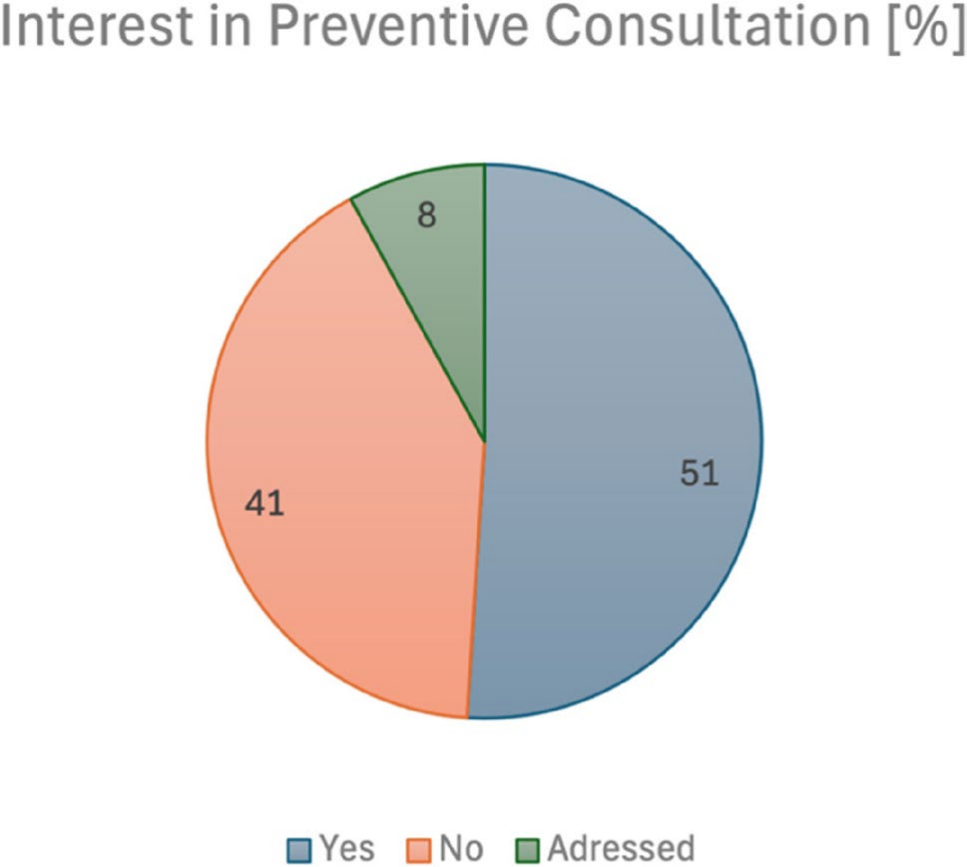
Interest in a preventive consultation about end-of-life care

Patients were asked if they would be interested in having preventive end-of-life care discussions with a doctor or healthcare worker. As shown in Fig. 6, 51% (*n* = 26) of patients responded positively to this question, while 41% (*n* = 21) expressed a lack of interest in a preventive consultation. Only 8% (*n* = 4) were found to have undergone such a discussion already. Although the majority of respondents (74.5%) were aware that bedriddenness and immobility are associated with an increased risk of death, only half of those surveyed (51%) said that they had become more interested in advance care planning as a result of the current situation and hospital treatment.

## Discussion

Immobilising fractures, such as those of the proximal femur, are considered to be common in geriatric patients [14]. A number of studies have shown that the prevalence of such fractures is expected to increase significantly in the future [5]. The postoperative course of proximal femoral fractures is associated with a high degree of permanent morbidity, reduced quality of life, a high mortality, and reduced mobility and independence [15, 16].

In contrast to cancer, very few patients are aware that immobilising fractures are also associated with a high mortality.

A study by Dunn et al. in 2016 examined the experience of geriatric patients with hip fractures and found that only 14% of patients had an end-of-life discussion during their hospital stay, while a significant proportion of 86% had no documented end-of-life discussion [17]. It is therefore essential that patients have thought about the end of life and have made their wishes known, so that family members and health care professionals can work towards fulfilling the patient’s wishes.

The purpose of having an advance directive or a power of attorney is to ensure that the patient’s wishes and ideas are carried out in the event that the patient is unable to give consent [18].

In our study, 84% of respondents had written at least an advance directive or a power of attorney, and 59% had written both. The prevalence of advance care documents in the present cohort was higher than in previous representative German population surveys [19].

The study also found that 56% of respondents who had thought about a preferred place of death had communicated this wish to a third person. A significant body of research, both national and international, has shown that the overwhelming preference of individuals is to die in the comfort of their own home, in accordance with their personal wishes [20–25]. This study shows, in line with similar research in oncology patients, that the majority of patients express a desire to die in their own home [11, 12]. In cancer patients, it has also been shown that patients prefer to remain at home as long as they are not seriously ill or dependent on help from others. Conversely, as symptom burden escalates and patients become more dependent on medical care, there is a shift in preference towards dying in a hospice, a preference that is often shared by family members [11, 12].

Research has consistently shown comparable results for those with chronic illness or living alone. Fereidouni’s seminal work described how changes in preferred place of death depend on demographic, disease-related and psychosocial variables [22, 26].

Another study highlighted that frailty, cognitive impairment and concerns about quality of life shape the framework for end-of-life decision-making [27].

Patients consistently cite maintaining cognitive function, maintaining close family relationships, regaining mobility and independence, and effective pain management as important goals of care. However, the low rate of specific advance care planning for immobilising fractures suggests that discussions focus on the acute illness rather than the specific complications associated with these injuries. Barriers that have been identified include clinician reluctance to engage in sensitive discussions, potential conflicts with treatment goals, and a mismatch between documented patient wishes and subsequent care decisions [17].

An advance care directive is a document designed to facilitate the implementation of the patient’s individual wishes and to avoid overtreatment in the last phase of life [28, 29].

Discussions about end-of-life care and the benefits that patients can gain from early care planning have been described by Brighton and Bristowe [30]. In the present study, 47% of respondents reported that they at least occasionally thought about their wishes for the end-of-life. However, only 25.5% of these individuals had been asked about their wishes, most of whom were asked by their children. Additionally, only 11% of respondents said that someone outside their family had asked them about their end-of-life wishes. In the context of inpatient trauma surgery admissions, only 4 patients reported having had a discussion with someone about advance care planning, although 50.9% of participants would have been interested in such a discussion.

The results of this study suggest that a proportion of patients may have a desire to discuss treatment and care options. This observation is in line with the results of previous studies conducted on patients with advanced cancer [31]. Despite the fact that approximately half of the patients surveyed do not currently wish to engage in such discussions, it is in the responsibility of the attending physician to determine during the patient consultation whether the patient has a need for a discussion about end-of-life care and requires support in preparing an advance directive or is even interested in advance care planning (ACP). The ACP process involves the formulation of a very detailed advance directive through discussion with a specialist. From the outset, ACP aims to encourage relatives to be present at these discussions in order to be informed about the patient’s wishes. This approach is based on the premise that it can help to avoid potential misunderstandings and misconceptions within the family unit [6, 32].

Another problem is that most physicians are not trained in end-of-life discussions. The “Serious Illness Conversation” (SIC) guide is a valuable resource for facilitating discussions about end-of-life care [33, 34]. End-of-life discussions should be offered to patients not just once, but throughout their lives. Patients and their relatives should be aware that any issues or changes in the patient’s wishes can be addressed at any stage of the care process.

### Limitations

The present study is limited by its single-centre design and the relatively small sample size and therefore cannot be considered representative. However, it should be noted that slightly more than half of the inpatients treated during the study period could not be included in the study due to reasons that made communication about the topic impossible. This is a noteworthy observation, as these individuals constitute the very demographic of patients who are no longer equipped to articulate their treatment preferences and end-of-life wishes. This aspect once again emphasises the need for early communication about patients’ desires. In addition, the questionnaire used in this study has not been formally validated. The results of the study cannot be extrapolated to the rest of Germany or other countries, as some aspects of communication between patients and their families and healthcare providers may be influenced by cultural factors and protocols specific to a healthcare system or workplace.

## Conclusion

Discussions concerning the end of life are seldom initiated. This paucity of discussion with patients can result in unwarranted overtreatment at the end of life. A significant proportion of patients express a desire to have end-of-life discussions with their physicians.

It is recommended that requests for advance care documents are made on a regular basis and that patients' wishes regarding end-of-life care are established. In cases where such documents are not prepared, healthcare providers are advised to emphasise the necessity for such documents and to offer assistance in their preparation (hospital staff as well as general practitioners).

## Data Availability

The data are available upon reasonable request from the authors.
